# Timing of reduction glossoplasty and clinical outcomes in children with Beckwith–Wiedemann syndrome

**DOI:** 10.3389/fped.2026.1937579

**Published:** 2026-09-30

**Authors:** Na-Kyum Park, Yoon Se Lee, Seung Hoon Han, Minsu Kwon, Young Ho Jung, Seung-Ho Choi

**Affiliations:** Department of Otorhinolaryngology–Head and Neck Surgery, Asan Medical Center, University of Ulsan College of Medicine, Seoul, Republic of Korea

**Keywords:** Beckwith–Wiedemann syndrome, macroglossia, malocclusion, oral feeding, reduction glossoplasty

## Abstract

**Background:**

Macroglossia in pediatric patients, particularly those with Beckwith–Wiedemann syndrome (BWS), can cause airway compromise, feeding difficulties, and dentofacial abnormalities. However, the optimal timing of reduction glossoplasty and its impact on clinical outcomes remain uncertain. This study aimed to evaluate the clinical outcomes of reduction glossoplasty in children with BWS, with particular emphasis on the association between age at surgery and postoperative outcomes.

**Methods:**

We retrospectively reviewed 21 pediatric patients with macroglossia who underwent reduction glossoplasty between 2017 and 2025 at a single institution. Patients were stratified into younger and older groups according to the median age at surgery (16.8 months). Associations between age at surgery and postoperative outcomes were analyzed.

**Results:**

All patients had BWS. Malocclusion occurred in 61.9% of patients and was significantly associated with older age at surgery (median 23.3 vs. 13.4 months, *P* = 0.004). The prevalence of malocclusion was significantly higher in the older group than in the younger group (90.9% vs. 30.0%, *P* = 0.012). Wound dehiscence occurred in 33.3% of patients but was managed conservatively in most cases, resulting in a reoperation rate of 14.3%. Age at surgery was not associated with postoperative complications (*P* = 0.799). Oral feeding was initiated at a median of 3.2 postoperative days and was delayed in patients who developed complications (*P* = 0.031).

**Conclusions:**

Reduction glossoplasty is a feasible treatment for children with BWS-associated macroglossia. Younger age at surgery was associated with a lower prevalence of malocclusion, whereas age at surgery was not significantly associated with postoperative complications. These findings support individualized surgical decision-making that balances potential dentofacial benefits against perioperative risks.

## 1 Introduction

Macroglossia is characterized by an enlarged tongue that often protrudes outside the mouth at rest (1). Congenital macroglossia commonly presents as part of a syndrome, such as Beckwith–Wiedemann syndrome (BWS), Down syndrome, and Klippel–Trenaunay–Weber syndrome (2). The incidence of macroglossia varies depending on the underlying etiology. BWS is an overgrowth disorder that classically presents with a triad of features, including macroglossia, abdominal wall defects, and gigantism. Up to 94% of patients with BWS exhibit macroglossia, which is attributed to muscle fiber hyperplasia and is therefore classified as true generalized macroglossia (3). Relative macroglossia is also commonly observed in individuals with Down syndrome, in whom the tongue may appear enlarged relative to the smaller dimensions of the oral cavity (4).

Some patients with mild macroglossia show spontaneous improvement with normal mandibular and skeletal growth, without the need for surgical intervention. However, macroglossia is a known predisposing factor for dental deformities, including mandibular prognathism and malocclusion, due to abnormal tongue positioning, dental arch misalignment, and jaw malformation (5–9). If left uncorrected, increased tongue volume may further contribute to speech and language impairments as affected children grow older (1, 2, 10).

Reduction glossoplasty is typically indicated when macroglossia results in one or more of the following problems: airway obstruction, feeding difficulties, persistent drooling, and cosmetic concerns (11). In addition, prophylactic reduction glossoplasty may be considered to prevent poor lip seal and impaired tongue mobility, which can lead to dysarticulation.

Surgical treatment aims to restore the size and shape of the tongue while preserving its function, including taste and sensation, swallowing, and articulation (6). Due to the rarity of macroglossia requiring surgical intervention, a variety of surgical techniques have been developed. The choice of reduction technique depends on the dimensions of tongue enlargement and the functional requirements of each patient. Anterior wedge resection primarily reduces tongue length, whereas central reduction is used to reduce tongue width and thickness (6, 11). The keyhole technique combines anterior wedge and central reduction, allowing simultaneous reduction in multiple dimensions while preserving the lateral neurovascular bundles (12). The standard keyhole pattern can be modified according to individual tongue morphology by adjusting the extent of anterior and central resection to achieve the desired reduction in length, width, and thickness.

However, several complications remain a concern, including bleeding, wound dehiscence, and insufficient reduction, which may necessitate additional resection to relieve symptoms. Furthermore, postoperative edema may compromise the airway in the immediate postoperative period. In the long term, patients may experience sensory or motor dysfunction (2, 13). Despite these potential complications, the benefits of reduction glossoplasty may justify surgical intervention in selected patients.

Recent studies have increasingly focused on the timing of tongue reduction surgery in children with Beckwith–Wiedemann syndrome (14–16). Tongue reduction has been associated with favorable functional outcomes (15), although the timing of surgery requires consideration of potential perioperative risks (16). Recent studies, including a systematic review, have further suggested that tongue reduction performed at an earlier age may be associated with more favorable dentofacial outcomes and a lower risk of malocclusion (14, 15). However, the optimal timing of surgery remains controversial because surgical indications, techniques, and outcome measures vary considerably across studies (15, 16). Nevertheless, evidence directly examining the association between age at surgery and both dentofacial outcomes and postoperative complications remains limited. Therefore, this study aimed to evaluate the clinical outcomes of reduction glossoplasty in children with BWS, with particular emphasis on the association between age at surgery and postoperative outcomes.

## 2 Materials and methods

### 2.1 Study population and design

We retrospectively reviewed 21 patients with macroglossia who underwent reduction glossoplasty between June 2017 and June 2025 at Asan Medical Center. Patients with mass lesions in the oral cavity, such as vascular tumors, lymphatic malformations, or venous malformations, were excluded. No patients met these exclusion criteria; therefore, all 21 patients were included in the final analysis. Because of the rarity of BWS-associated macroglossia requiring reduction glossoplasty, no *a priori* sample size calculation was performed. All consecutive eligible patients treated during the study period were included. This study was approved by the Institutional Review Board of Asan Medical Center (2024-0004), and the requirement for informed consent was waived.

Although the indications for surgery are often not clearly defined and the timing of surgical intervention may appear arbitrary, in our study, surgical resection was indicated when the enlarged tongue protruded beyond the lips at rest and was expected to cause malocclusion, feeding difficulties, or airway compromise. Although some patients presented with respiratory symptoms such as dyspnea, no patient required surgery for acute airway compromise. Surgical decisions were made in consultation with a pediatric dentist, and all procedures were performed by a single surgeon.

The surgical technique was individualized according to the degree and dimensions of tongue enlargement. The extent and pattern of resection were adjusted to achieve the required reduction in tongue length, width, and thickness based on the preoperative tongue morphology and presenting functional problems (Figure 1). Anterior wedge resection was primarily used when reduction in tongue length was required, whereas central reduction was used when reduction in tongue width and thickness was required. The keyhole technique, combining anterior wedge and central reduction, was used when multidimensional reduction was required. The extent of each component was modified according to the individual tongue morphology and the desired degree of reduction. This approach allowed preservation of the lateral neurovascular bundles while enabling substantial three-dimensional debulking of the tongue (6).

**Figure 1:**
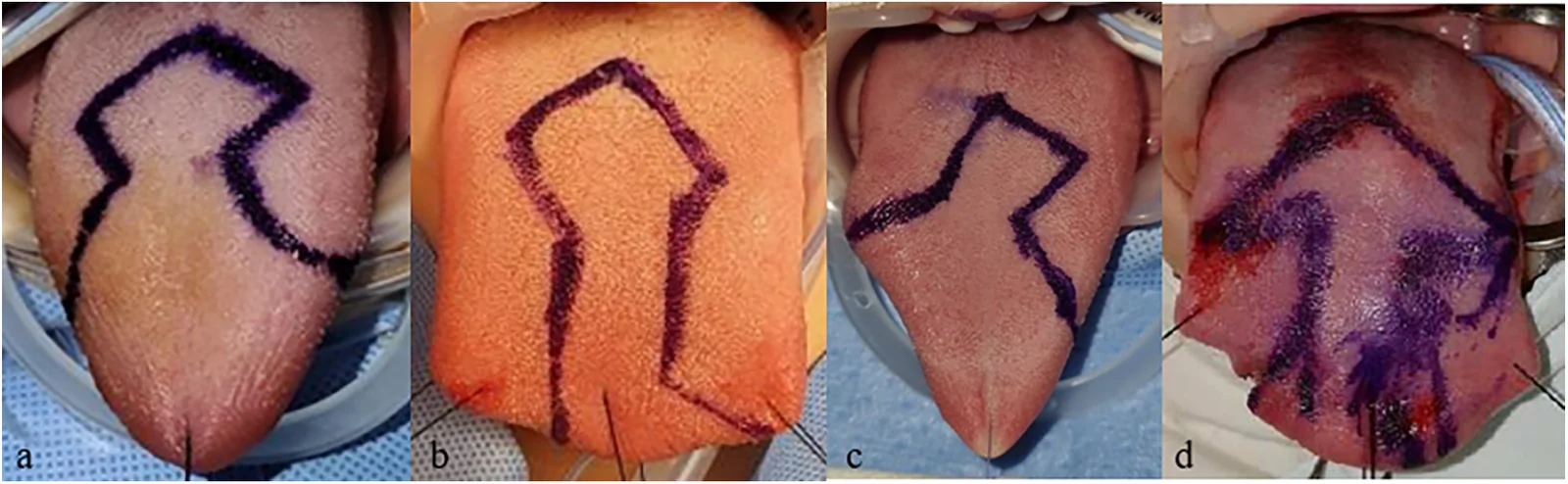
Techniques of reduction glossoplasty. **(a)** Marking of the standard keyhole incision pattern. **(b–d)** The modified keyhole techniques for each patient.

Demographic data, including age at surgery, sex, associated craniofacial anomalies, and presenting symptoms, were collected. To assess growth status, weight and height at the time of surgery were compared with the 2017 Korean National Growth Charts (17). The following perioperative data were reviewed: surgical technique, operative time, length of hospital stay, intensive care unit (ICU) admission, postoperative feeding methods (e.g., oral feeding, nasogastric tube feeding), and complications. Postoperative satisfaction was assessed retrospectively based on parental reports documented during postoperative outpatient visits. Satisfaction was determined according to the parents' subjective assessment of improvement in presenting symptoms and the cosmetic appearance of the tongue. No formal validated questionnaire or standardized satisfaction scale was used.

Postoperative complications were further classified as minor (resolved with conservative management) or major (requiring reoperation). Postoperative occlusal status was clinically assessed during follow-up in the pediatric dental clinic, including evaluation for progression of malocclusion. Malocclusion was determined based on clinical assessment by pediatric dentists and documentation in the medical records. No standardized quantitative occlusal measurements were retrospectively available for all patients. Treatment outcomes were evaluated based on clinical outcomes, including postoperative complications, feeding status, and changes in presenting symptoms, as assessed by a multidisciplinary team consisting of the surgeon, pediatricians, dentists, and speech-language therapists.

### 2.2 Statistical analysis

Patients were divided into a younger group (*n* = 10) and an older group (*n* = 11) based on the median age at surgery (16.8 months). The prevalence of malocclusion and postoperative complications was compared between the two groups using Fisher's exact test.

Age at surgery was compared between patients with and without malocclusion and between patients with and without postoperative complications using the Mann–Whitney *U* test. Time to initiation of oral feeding was also compared between patients with and without postoperative complications using the Mann–Whitney *U* test.

Statistical analyses were performed using SPSS version 22.0 (IBM Corp., Armonk, NY, USA). A *P* value <0.05 was considered statistically significant.

## 3 Results

### 3.1 Demographic data

Table 1 summarizes the demographic and clinical characteristics of the 21 patients. Fifteen patients were female (71.4%). All patients were diagnosed with Beckwith–Wiedemann syndrome, and two patients had concomitant cleft palate. The median age at surgery was 16.8 months (range, 5.0–112.4 months).

**Table 1:** Demographic and clinical characteristics of patients (*n* = 21).

Variable	Values
Sex, *n* (%)	
Female	15 (71.4)
Male	6 (28.6)
Age at surgery (months)	16.8 (5.0–112.4)
Presenting symptoms, *n* (%)	
Isolated tongue protrusion	12 (57.1)
Malocclusion	13 (61.9)
Dyspnea	4 (19.0)
Snoring/sleep apnea	3 (14.3)
Feeding difficulties	3 (14.3)
Drooling	2 (9.5)
Surgical technique, *n* (%)	
Keyhole technique	17 (81.0)
Central reduction	2 (9.5)
Anterior wedge resection	2 (9.5)
Operative time (minutes)	30 (18–68)
Length of hospital stay (days)	5 (3–12)
Postoperative complications, *n* (%)	
Wound dehiscence (total)	7 (33.3)
Conservative management	4 (19.0)
Revision glossoplasty	3 (14.3)
Other complications	0 (0.0)

Values are presented as number (%) or median (range). Other complications include infection, bleeding, and airway obstruction.

Twelve patients (57.1%) presented with isolated tongue protrusion, while the remaining patients had additional symptoms, including malocclusion (61.9%), snoring or sleep apnea (14.3%), feeding difficulties (14.3%), dyspnea (19.0%), and drooling (9.5%) (Table 1). According to the 2017 Korean National Growth Charts, 13 patients (61.9%) were classified as overweight and 8 patients (38.1%) had normal weight at the time of surgery. Height was within the normal range in most patients; however, one patient (4.8%) had short stature, with a height percentile of 0.2. Preoperatively, all patients demonstrated tongue protrusion accompanied by persistent mouth opening at rest, reflecting the inability of the enlarged tongue to be adequately accommodated within the oral cavity.

Patients with malocclusion were significantly older at the time of surgery than those without malocclusion (median 23.3 vs. 13.4 months, *P* = 0.004) (Figure 2). When patients were stratified by the median age, the prevalence of malocclusion was significantly higher in the older group than in the younger group (90.9% vs. 30.0%, *P* = 0.012) (Table 2).

**Figure 2:**
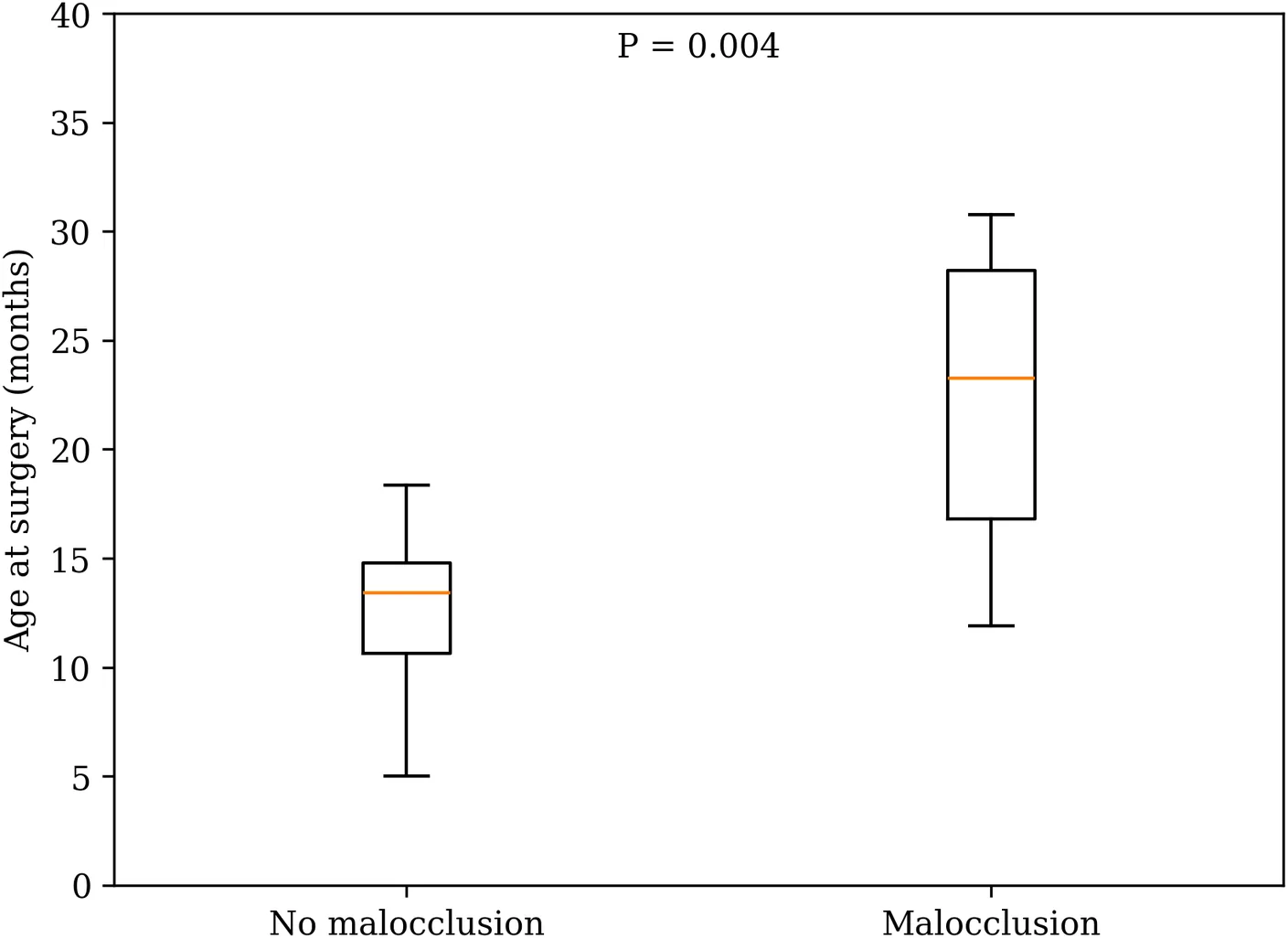
Box-and-whisker plot of age at surgery according to the presence of malocclusion. Patients with malocclusion were significantly older at the time of surgery than those without malocclusion (*P* = 0.004).

**Table 2:** Comparison of malocclusion and postoperative complication rates according to median age at surgery.

Outcome	Younger group (≤16.8 months, *n* = 10)	Older group (>16.8 months, *n* = 11)	*P*
Malocclusion, *n* (%)	3 (30.0)	10 (90.9)	0.012
Complication, *n* (%)	3 (30.0)	4 (36.4)	1.000

*P* values were calculated using Fisher's exact test.

Regarding surgical techniques, the keyhole technique was used in 17 patients (81.0%), followed by central reduction (9.5%) and anterior wedge resection (9.5%) (Table 1). The median operative time was 30 min (range, 18–68 min).

### 3.2 Hospital stay and postoperative feeding

The median length of hospital stay was 5 days (range, 3–12 days). Most patients were directly admitted to the general ward after reduction glossoplasty. Only one patient required admission to the intensive care unit (ICU) due to concurrent Bochdalek hernia repair.

Oral feeding was initiated at a median of 3.2 days postoperatively (range, 1–7 days). Eight patients (38.1%) did not require nasogastric tube feeding and were able to start oral feeding on the first postoperative day.

The longest duration of nasogastric tube use was 7 days. Two patients required nasogastric tube feeding for 7 days; one underwent reoperation on postoperative day 4, whereas the other underwent concurrent hernia repair. These clinical conditions necessitated prolonged use of enteral feeding.

### 3.3 Treatment outcomes

Following glossoplasty, reduction of tongue volume allowed repositioning of the tongue within the oral cavity. As a result, patients demonstrated improved mouth closure at rest during postoperative evaluation. Based on parental reports documented during postoperative outpatient visits, 20 patients (95.2%) were considered to have satisfactory outcomes in terms of symptom improvement and cosmetic appearance. One patient underwent additional glossoplasty because of parental dissatisfaction with the cosmetic appearance, despite sufficient volume reduction and improvement in functional symptoms. All patients were followed up in a pediatric dental clinic for evaluation of occlusion, and no clinically significant progression of malocclusion was documented during the available follow-up period.

During a median follow-up period of 1 year (range, 0.5–5 years), postoperative complications occurred in 7 patients (33.3%), all of which were wound dehiscence (Table 1). Of the seven patients with wound dehiscence, five had undergone the keyhole technique, one anterior wedge resection, and one central reduction. Wound dehiscence was managed conservatively in four patients, while three patients required revision surgery; all three patients requiring revision had undergone the keyhole technique. There were no cases of surgical site infection or airway compromise, and no patient required tracheostomy or prolonged intubation.

There was no significant association between age at surgery and postoperative complications (*P* = 0.799). When patients were stratified by the median age, there was no significant difference in postoperative complication rates between the younger and older groups (30.0% vs. 36.4%, *P* = 1.000) (Table 2).

The initiation of oral feeding was significantly delayed in patients who developed complications compared with those who did not (median 5.0 vs. 1.5 days, *P* = 0.031) (Figure 3).

**Figure 3:**
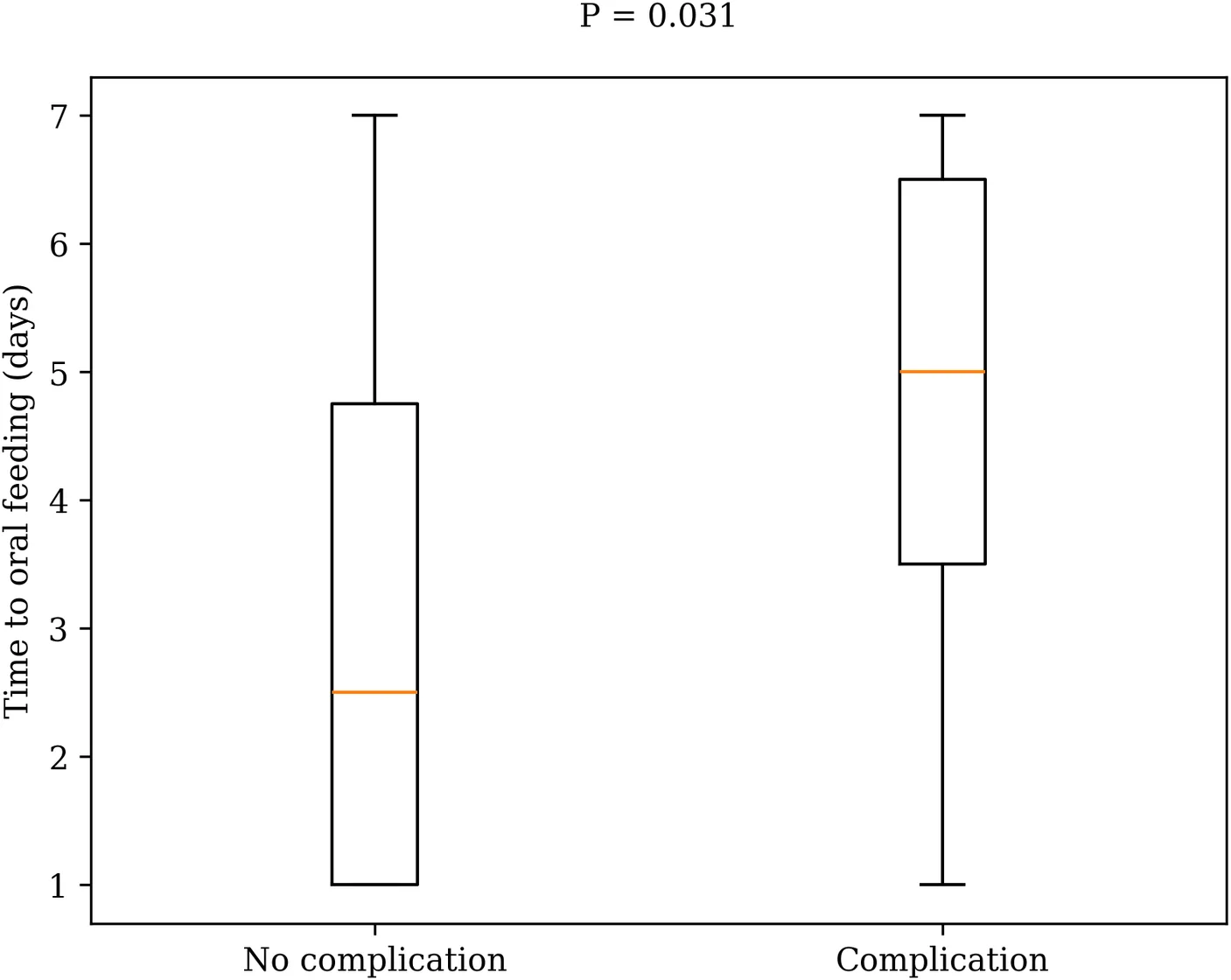
Box-and-whisker plot of time to initiation of oral feeding according to postoperative complications. Patients with complications showed significantly delayed initiation of oral feeding compared with those without complications (*P* = 0.031).

## 4 Discussion

Reduction glossoplasty is performed in children with BWS to alleviate functional problems caused by macroglossia and to reduce its potential adverse effects on dentofacial development. Previous studies have reported favorable functional and dentofacial outcomes following tongue reduction surgery (15, 18–20). However, the optimal timing of surgery remains controversial, particularly because the potential dentofacial benefits of earlier intervention must be considered alongside perioperative safety (14–16).

In the present study, we evaluated the clinical outcomes of reduction glossoplasty in 21 children with BWS, with particular emphasis on age at surgery. Patients with malocclusion were significantly older at the time of surgery than those without malocclusion, and the prevalence of malocclusion was higher in the older group. In contrast, age at surgery was not significantly associated with postoperative complications. Wound dehiscence was the only postoperative complication observed and was managed conservatively in most cases, with three patients requiring revision surgery. Oral feeding was initiated at a median of 3.2 days postoperatively and was delayed in patients who developed complications. These findings suggest that surgical timing may be relevant to dentofacial outcomes, while an association between younger age at surgery and increased postoperative complications was not observed in this cohort.

Persistent tongue protrusion may adversely affect dentofacial development through abnormal mechanical forces on the dentoalveolar structures. Abnormal tongue position and persistent open-mouth posture have been associated with altered dental arch formation and malocclusion (21). Reduction of tongue volume may improve accommodation of the tongue within the oral cavity and restore lip competence, thereby reducing a functional factor that may contribute to malocclusion. In our cohort, patients with malocclusion were significantly older at the time of surgery than those without malocclusion, and the prevalence of malocclusion was higher in the older group. These findings are consistent with recent studies suggesting that earlier tongue reduction may be associated with more favorable dentoskeletal outcomes in children with BWS (14, 15). However, the median age of 16.8 months used for group stratification in our study was an exploratory cutoff based on the distribution of our cohort and should not be interpreted as an optimal age for surgery. Furthermore, because malocclusion was already present at the time of surgery, the retrospective association between younger age and a lower prevalence of malocclusion cannot establish a preventive effect of earlier surgery.

The potential dentofacial benefits of earlier intervention must also be balanced against perioperative safety. Recent evidence has suggested that surgery at a very young age may be associated with an increased risk of perioperative complications (16). In contrast, we found no significant association between age at surgery and postoperative complications, either when age was analyzed as a continuous variable or when patients were stratified according to the median age. Nevertheless, given the small number of patients and complications in our cohort, these findings should not be interpreted as evidence of equivalent safety across different ages. Taken together, our findings support individualized determination of surgical timing based on the severity of macroglossia, functional impairment, dentofacial development, and potential perioperative risks rather than on a fixed age cutoff.

The optimal timing of oral feeding after reduction glossoplasty also remains an important aspect of postoperative management because of concerns regarding wound dehiscence, bleeding, and infection (2, 11, 13). In our cohort, oral feeding was initiated significantly later in patients who developed postoperative complications than in those without complications. However, the timing of oral feeding was determined according to postoperative clinical conditions rather than by a standardized protocol, making it difficult to determine the direction of this association. Therefore, our findings do not establish whether earlier initiation of oral feeding influences the risk of wound dehiscence. Notably, although wound dehiscence occurred in seven patients, four cases were successfully managed conservatively and only three required revision surgery. Further prospective studies using standardized postoperative feeding protocols are needed to clarify the relationship between feeding timing and wound healing after reduction glossoplasty.

The surgical technique should be individualized according to the anatomical characteristics of macroglossia and the functional requirements of each patient (22). In our cohort, the keyhole technique was used in the majority of patients (81.0%), reflecting the frequent need for multidimensional reduction of tongue length, width, and thickness. This technique enables substantial tongue reduction while preserving the lateral neurovascular bundles (12). Nevertheless, one patient required additional glossoplasty because of parental dissatisfaction with the cosmetic appearance despite adequate volume reduction and functional improvement. These findings emphasize that surgical planning should consider not only the dimensions of tongue enlargement and functional impairment but also cosmetic expectations. Thorough preoperative counseling regarding the anticipated functional and cosmetic outcomes is therefore important.

An important aspect of the present study is the simultaneous evaluation of age at surgery in relation to both malocclusion and postoperative complications. This approach provides additional clinical data relevant to balancing the potential dentofacial benefits of earlier tongue reduction against perioperative risks when determining surgical timing. Nevertheless, several limitations should be acknowledged. First, this was a retrospective, single-center study with a relatively small sample size, which limited statistical power, particularly for the analysis of postoperative complications, and may limit the generalizability of our findings. In addition, the small number of patients and the predominance of the keyhole technique precluded meaningful comparison of technique-specific complication rates, while the retrospective data did not allow systematic assessment of the potential effects of suture selection or patient compliance on wound dehiscence. Second, the association between age at surgery and malocclusion was cross-sectional because malocclusion was assessed at the time of surgery; therefore, a causal preventive effect of earlier surgery cannot be established. Third, the median age of 16.8 months was used for exploratory group stratification and should not be interpreted as a clinically validated cutoff for surgical timing. Fourth, longitudinal dentofacial changes were not assessed using standardized quantitative measurements, limiting our ability to determine the effect of surgery on subsequent occlusal development. In addition, parental satisfaction was assessed retrospectively without a validated questionnaire, and the duration of postoperative follow-up varied among patients. Larger prospective studies with standardized dentofacial assessments and long-term follow-up are needed to better define the optimal timing of reduction glossoplasty in children with BWS.

In conclusion, reduction glossoplasty is a feasible treatment for children with BWS-associated macroglossia. In our cohort, younger age at surgery was associated with a lower prevalence of malocclusion, whereas age at surgery was not significantly associated with postoperative complications. These findings suggest that surgical timing should be individualized by balancing potential dentofacial benefits against perioperative risks rather than relying on a fixed age cutoff. However, because of the retrospective design and small sample size, a causal preventive effect of earlier surgery cannot be established. Larger prospective studies with standardized dentofacial assessments and long-term follow-up are needed to determine the optimal timing of reduction glossoplasty and its long-term effects on occlusal development and tongue function.

## Data Availability

The datasets presented in this article are not readily available because of patient privacy and institutional regulations. Requests to access the datasets should be directed to the corresponding author.
